# Levels of brain natriuretic peptide are associated with peripheral arterial disease in subjects with type-2 diabetes mellitus

**DOI:** 10.1186/1472-6823-14-27

**Published:** 2014-03-21

**Authors:** Qi-hui Jin, Wan-lan Ye, Huai-hong Chen, Xiao-jun He, Tian-lang Li, Qiang Liu, Liang Zhong, Lei Xu, Chun-mao Han

**Affiliations:** 1 Geriatric Medicine, The Second Affiliated Hospital of Zhejiang University School of Medicine, 88 JieFang Rd, Hangzhou, Zhejiang 310009, China; 2 Endocrinology, The Second Affiliated Hospital of Zhejiang University School of Medicine, 88 JieFang Rd, Hangzhou, Zhejiang 310009, China; 3 Emergency Medicine, The Second Affiliated Hospital of Zhejiang University School of Medicine, 88 JieFang Rd, Hangzhou, Zhejiang 310009, China; 4 Department of Burn, The Second Affiliated Hospital of Zhejiang University School of Medicine, 88 JieFang Rd, Hangzhou, Zhejiang 310009, China

**Keywords:** Brain natriuretic peptide, Peripheral arterial disease, Type-2 diabetes mellitus

## Abstract

**Background:**

The effects of brain natriuretic peptide (BNP) on the risk of cardiovascular disease and atherosclerosis have been studied. However, little information is available regarding peripheral arterial disease (PAD), particularly among subjects with type-2 diabetes mellitus (T2DM). The aim of our study was to assess the potential relationship between BNP levels and PAD among T2DM patients.

**Methods:**

The study cohort was 507 T2DM outpatients in which BNP levels were measured. Cross-sectional associations between BNP levels (in tertiles) and PAD were examined.

**Results:**

Compared withT2DM patients without PAD, BNP levels were markedly higher in patients with PAD (*p* = 0.001). Correlation analyses showed that the BNP level was negatively correlated with the ankle–brachial index (r = −0.453, *p* = 0.033). At a cutoff value of 78.2 pg/ml, the BNP level showed a sensitivity of 71.9%, a specificity of 68.1%, and a positive predictive value of 84.3% for a diagnosis of PAD. The area under the receiver-operating characteristic curve increased significantly if BNP levels were incorporated into a predictive model of the potential risk factors for PAD (0.85 *vs* 0.81, *p* = 0.029).

**Conclusions:**

BNP is a potential and promising biomarker for PAD screening in T2DM patients.

## Background

Peripheral arterial disease (PAD) is a subclinical measure of atherosclerotic vascular disease and a strong independent risk factor for cardiovascular disease (CVD) and mortality. Type-2 diabetes mellitus (T2DM) is associated with accelerated atherosclerosis and an increased risk of PAD. The incidence of PAD in T2DMpatients is high [1]. Patients with T2DM have a fourfold increased risk of PAD [2], and the prevalence of PAD is higher in diabetic than in non-diabetic populations [3].

Brain natriuretic peptide (BNP) is secreted predominantly from the ventricular myocardium and is a useful predictor of cardiovascular disease risk [4]. Interestingly, increasing evidences show that BNP is a useful marker not only for cardiac function, but also for other vasculopathies. Increased BNP concentrations have been associated with atherosclerosis [5], and their levels been shown to be elevated in a general population with PAD [6-8].

The development of PAD is associated with several risk factors and multiple biomarkers representing various etiologic pathways of atherosclerosis [9]. Recent findings suggest a close relationship between glucose metabolism and BNP levels. BNP levels were found to be higher in patients with T2DM and inversely associated with the risk of T2DM [10-12], though the mechanism is not known. These observations suggested a potential role of BNP in the development of T2DM with PAD. However, it is not clear if BNP contributes to reducing or improving the morbidity of PAD in T2DM. To explore such a possibility, we measured serum BNP levels and assessed the potential relationship between BNP levels and PAD among outpatients with T2DM.

## Methods

### Study patients

The study was approved by the Ethics Committee of the Second Affiliated Hospital of Zhejiang University (Zhejiang, China). Written informed consent was obtained from all subjects to participate in this study.

A total of 507 outpatients with T2DM (age ≥18 years) in the Department of Endocrinology of the Second Affiliated Hospital of Zhejiang University from January 2012 to January 2013 were recruited. Inclusion criteria were a diagnosis of T2DM according to criteria set by the American Diabetes Association [13] and PAD was defined as arterial insufficiency with an ankle–brachial index(ABI) ≤0.90 in either leg [14]. Exclusion criteria were:severe infection (ischemic ulceration/necrosis); acute or severe and chronic diabetic complications (diabetic ketoacidosis or coma, diabetic nephropathy, clinical albuminuria of diabetic nephropathy); poor glucose control (hemoglobin A1c > 10.0%) or requirement of insulin treatment; diseases of the liver or kidney; cancer or autoimmune disease; CVD (including cardiac systolic and diastolic dysfunction, valvular abnormalities, myocardial infarction, or cardiomyopathy, other structural heart diseases and changes in the electrocardiogram due to myocardial ischemia and various arrhythmias); use of diureticsor nitrates; any other condition that (in the investigator’s judgment) could affect study participation or confound data interpretation.

### General clinical data and laboratory measurements

Clinical and laboratory data were collected from medical records. Cardiac contraction and diastolic functions were evaluated by echocardiography. Diabetic neuropathy was examined by testing vibration (using a 128-Hz tuning fork), pin-prick sensation (using Neurotip™; Owen Mumford, Chipping Norton, UK), temperature sensation (warm and cool rods), and Achilles tendon reflex (tendon hammer). Albumin levels were measured in a spot urine sample, which was collected as the first void in the morning or at random. The results of albumin measurements in spot collections may be expressed as the urinary albumin-to-creatinine ratio (UACR). Albuminuria was defined as two of three the UACR of ≥30 μg/mg on at least two occasions within 3–6 months [15]. Demographic information (age, sex, blood pressure (BP), the body mass index (BMI) and duration of T2DM) was obtained at study baseline. Venous blood samples were collected from all patients in the morning after fasting for 10 h and stored at room temperature. Biochemical parameters such as levels of triglyceride (TG), total cholesterol (TC), high-density lipoprotein-cholesterol (HDL-C), low-density lipoprotein-cholesterol (LDL-C), C-reactive protein (CRP), creatinine, uric acid, and homocysteine were measured using an automated clinical chemistry analyzer (Advia 2400; Siemens, Munich, Germany). Hemoglobin A1c (HbA1c) levelswere measured by high-performance liquid chromatography (Variant II; Bio-Rad, Hercules, CA, USA).

### BNP peptide assay

Serum BNP levels were quantified using an electrochemiluminescence immunoassay (Advia Centaur XP; Siemens) with minimum and maximum detectable concentrations of 4.5 pg/ml and 5000 pg/ml, respectively (normal reference range <100 pg/ml). Tests were undertaken in our clinical laboratory. Inter- and intra-batch coefficients of variation were maintained within 5.5% and 3.5%, respectively.

### Measurement of the ABI

Measurement of the ABI is a validated, useful and easy tool to diagnose PAD. ABI measurements were done with the patient in the supine position. BP was measured in the bilateral brachial and dorsalis pedis arteries with an 8-MHz Doppler probe. The ABI was calculated by dividing the value of the systolic blood pressure (SBP) in the right or left ankle by the value of the SBP in the arm.

### Definitions

An ABI ≤0.9 was selected as the cutoff value for the diagnosis of PAD; an ABI >1.3 was selected as the cutoff value for calcification [14]. Hypertension was defined as SBP >140 mmHg, or diastolic BP >90 mmHg, or use of antihypertensive medications [16]. The diagnostic criteria of systolic dysfunction (left ventricular ejection fraction (EF) <50%) and diastolic dysfunction (E/A ratio <1or >2) were according to guidelines set by the European Society of Cardiology for the diagnosis and treatment of acute and chronic heart failure [17]. The diagnosis of diabetic neuropathycan be made after a careful clinical examination [18]. The diagnosis of clinical albuminuria was albuminuria ≥300 μg/mg [15]. Hyperlipidemia was defined as serum TC >6.2 mmol/l and/or TG >2.3 mmol/l or HDL-C <1.04 mmol/l (men), <1.17 mg/dl (women), or use of lipid-lowering agents [19].

### Statistical analyses

Data are the mean ± standard deviation (SD) or percentage (%) and analyzed using the Student’s *t*-test or chi-square test. Age, sex, CRP level, smoking, hypertension, hyperlipidemia, and duration of T2DM account for most of the risk associated with the development of PAD [20].

BNP levels did not have a normal distribution, and were divided into four quartiles for analyses: <26, 26–76, 76–100, and 100–529 pg/ml. Analysis of variance with the Bonferroni correction was used to compare mean baseline values among the four groups. Logistic regression models were fitted to calculate the odds ratio (OR) and 95% confidence interval (CI) for PAD. The association between serum BNP levels and PAD was analyzed using multiple regression analysis adjusted for other CVD risk factors. BNP levelswere highly skewed, so log_10_-transformed values were used to determine linear correlation coefficients for the association between BNP levelsand the ABI. The predictive value of BNP levels was assessed by analyses of receiver operating characteristic (ROC) curves. Area under the ROC curve was used to compare the accuracy of the ability to assess the likelihood of PAD between the models adjusted for potential risk factors with and without BNP levels.

Data were analyzed using SPSS v15.0 (SPSS, Chicago, IL, USA). Statistical assessments were two-tailed and *p* < 0.05 considered significant.

## Results

A total of 507 T2DM patients (324 malesand 183 females; mean age, (60.2 ± 7.2)years) were involved in this study. The median BNP level was 73 (interquartile range, 4.5–529) pg/ml. A total of 138 patients (27.2%) had PAD.

Patients were placed into two groups based on PAD and their characteristics are summarized in Table 1. The median BNP level was significantly higher in the PAD group than in the non-PAD group (78 [4.5–529] pg/ml vs. 71 [5.0–497] pg/ml, *p* = 0.001). Compared with the non-PAD group, the duration of T2DM and HbA1c were significantly greater in the PAD group (*p* = 0.035 and 0.034, respectively). No differences were shown with respect to age, BP, EF, E/A ratio, smoking habits, the BMI, the UACR, serum creatinine as well as levels of CRP, uric acid, fibrinogen, LDL-C, HDL-C and TC (*p* > 0.05 for all) between the two groups.

**Table 1:** Baseline characteristics of PAD (ABI ≤ 0.9) and non–PAD (1.3 ≥ ABI > 0.9) diabetic patients

**Variables**	**PAD n = 138**	**No-PAD n = 369**	***p *** **value**
Male/Female, n	103/35	260/109	0.414
Age (years)	60.9 ± 8.2	59.8 ± 7.7	0.161
BMI	20.22 ± 2.79	20.18 ± 2.66	0.882
Duration of diabetes (year)	6.3 ± 3.7	5.8 ± 3.2	0.035
ABI	0.78 ± 0.11	1.09 ± 0.12	0.000
Systolic blood pressure (mmHg)	132.4 ± 12.7	131.7 ± 11.5	0.554
Diastolic blood pressure (mmHg)	77.7 ± 6.7	78.1 ± 7.4	0.579
HbA1c (%)	7.9 ± 0.9	7.6 ± 0.8	0.034
Serum creatinine (μmol/l)	91.4 ± 7.2	90.9 ± 6.6	0.418
Serum uric acid (μmol/l)	234.7 ± 34.4	229.4 ± 32.7	0.105
UACR (μg/mg)	72.4 ± 27.2	68.5 ± 25.4	0.132
Total cholesterol (mmol/l)	4.87 ± 1.04	4.76 ± 1.11	0.313
Triglycerides (mmol/l)	1.24 ± 0.32	1.21 ± 0.27	0.291
High-density lipoprotein cholesterol (mmol/l)	1.09 ± 0.21	1.12 ± 0.22	0.167
Low-density lipoprotein cholesterol (mmol/l)	2.44 ± 0.43	2.39 ± 0.38	0.204
C-reactive protein (mg/l)	5.3 ± 1.1	5.2 ± 1.1	0.432
Fibrinogen (mg/dl)	3.21 ± 0.87	3.14 ± 0.79	0.388
Left ventricular ejection fraction (%)	61.7 ± 4.6	62.3 ± 5.1	0.227
E/A	1.22 ± 0.24	1.25 ± 0.26	0.547
BNP (median and interquartile range) (pg/ml)	78 (4.5–529)	71 (5.0–497)	0.001
Hypertension, n (%)	56 (40.58)	137 (37.13)	0.542
Hyperlipidemia, n (%)	58 (42.03)	129 (34..96)	0.142
Current smoking, n (%)	55 (39.86)	141 (38.21)	0.814
RAAS blockade, n (%)	32 (23.19)	81 (21.95)	0.859
Calcium channel blockers, n (%)	21 (15.22)	62 (16.80)	0.768
Statin therapy, n (%)	27 (19.57)	81 (21.95)	0.644
Aspirin therapy, n (%)	33 (23.91)	102 (27.64)	0.464

Date is expressed as mean ± standard deviation.

BMI, body mass index. ABI, ankle-brachial index. UACR, urinary albumin-to-creatinine ratio. BNP, brain natriuretic peptide. RASS, renin-angiotensin-aldosterone system. E, late diastolic filling velocity. A, early diastolic filling velocity.

**Figure 1:**
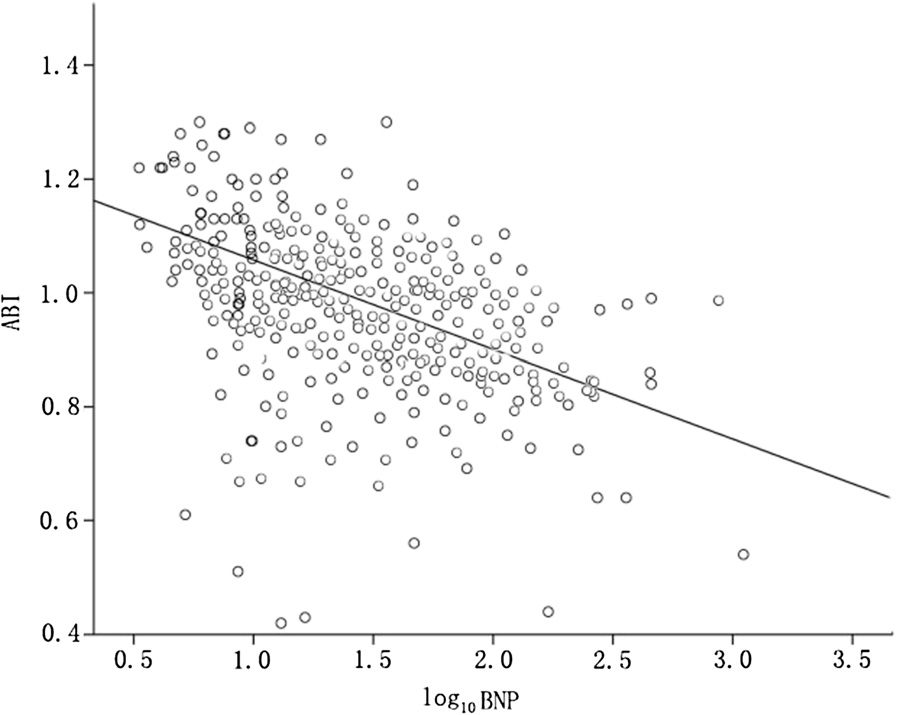
**The relationship between log**_**10**_**BNP and ABI (r = −0.453, *p*** **= 0.033).**

The BNP level was negatively correlated with the ABI (r = −0.453, *p* = 0.033) (Figure 1). Accordingly, we determined the significance and magnitude of the association between the BNP level and PAD. In model 1, after adjustment for age and sex, each 1-SD increment in the BNP level was associated with an increased risk of PAD (OR, 1.21; 95% CI, 1.19–1.45; *p* = 0.012). In model 2, after adjustment for age, sex, the BMI, BP, smoking habit, the UACR, duration of T2DM, as well as levels of uric acid, TG, LDL-C, HDL-C,and HbA1c, the trend remained significant (OR, 1.16; 95% CI, 1.04–1.34; *p* = 0.021). The odds of having PAD at baseline increased significantly with increase in the quartile of the BNP level (Table 2).

**Table 2:** Logistic regression analysis examining BNP quartiles in relation to prevalence of PAD in diabetic patients (n = 507)

**Group**	**Quartiles of BNP**		
	**OR**	**95% CI**	***p***
Model 1 with quartile group as categorical variables			
Group 1 (lowest values)	Reference		
Group 2	1.13	0.97–1.31	0.062
Group 3	1.19	1.11–1.34	0.018
Group 4 (highest values)	1.31	1.21–1.59	0.011
*P* for trend	1.22	1.17–1.41	0.015
1 SD change in BNP included as continuous variable	1.21	1.19–1.45	0.012
Model 2 with quartile group as categorical variables			
Group 1 (lowest values)	Reference		
Group 2	1.07	0.95–1.21	0.081
Group 3	1.15	1.02–1.31	0.047
Group 4 (highest values)	1.25	1.10–1.43	0.032
*P* for trend	1.18	1.11–1.35	0.039
1 SD change in BNP included as continuous variable	1.16	1.04–1.34	0.021

Model 1 was adjusted for age and sex.

Model 2 was adjusted for age, sex, BMI, BP, uric acid, UACR, smoking, TG, LDL-C, HDL-C, duration of diabetes and HbA1c.

BNP levels were as follows median (25–75% interquartile range): quartile 1, 24.0 (4.5 –25.0) pg/ml; quartile 2, 43.0 (26.5 –75.0) pg/ml; quartile 3, 86.0 (76.5 –100) pg/ml; quartile 4, 146.2 (100.5 –529) pg/ml.

**Figure 2:**
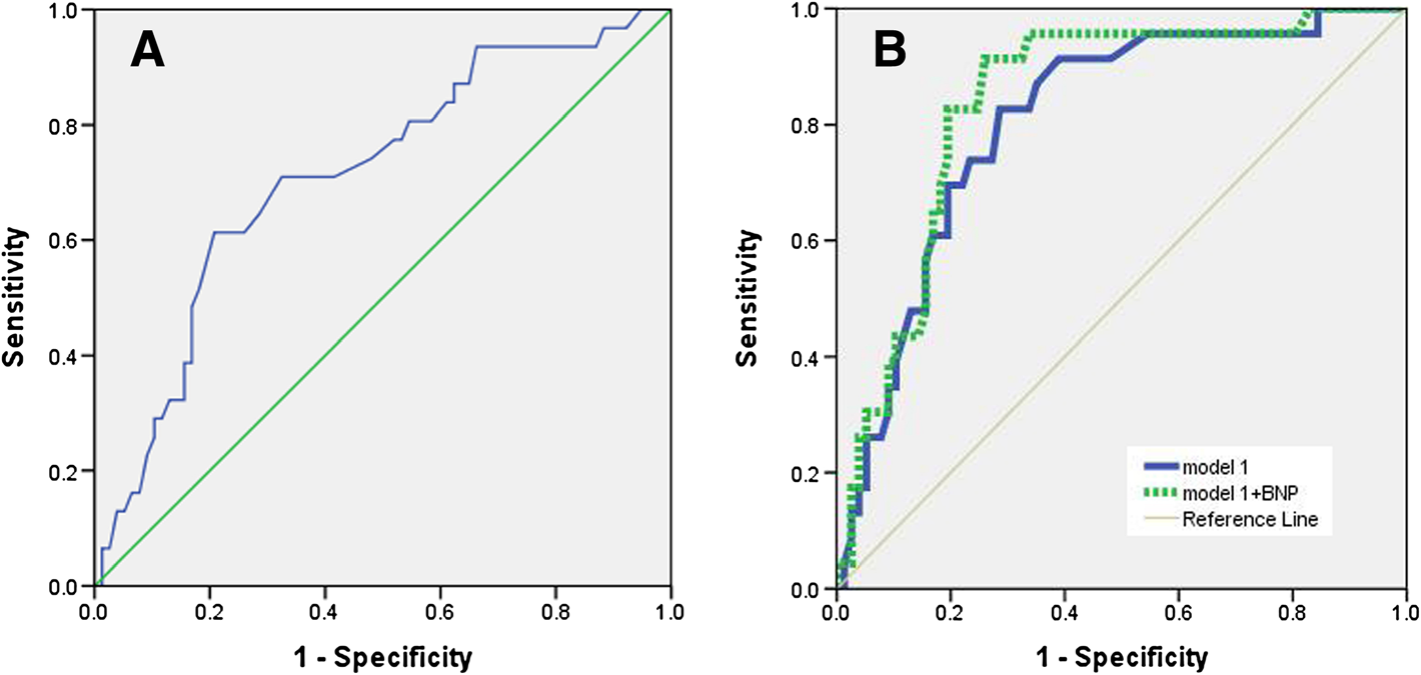
**The predictive value of BNP levels for PAD can be reflected in ROC plots. A.** BNP levels could be of use as predictive marker for PAD. **B.** Comparison of the assessment of the likelihood of the presence of PAD between models with or without BNP.

In the ROC analysis, BNP yielded an area under the curve (AUC) of 0.68 (95% CI, 0.62–0.77; *p* =0.008) for detection of PAD. A BNP level of 78.2 pg/ml was determined as the cutoff value that gave the best combination of sensitivity and specificity (0.719 and 0.681, respectively) (Figure 2A). We determined the effect of the BNP level on assessment of PAD by comparing the areas under the ROC curves between risk models with and without the BNP level (Figure 2B). The AUC associated with the ROC analysis of model 1 (including age, sex, the BMI, smoking habit, the UACR, hypertension, dyslipidemia, duration of T2DM,as well as levels of CRP, uric acid, and HbA1c) was 0.81 (95% CI, 0.74–0.87). Addition of the BNP level increased the AUC to 0.85 (95% CI, 0.77–0.91) (*p* =0.029 compared with model 1).

## Discussion

In the present study, we found a clear association between the BNP levels and the risk of PAD in T2DM patients without overt CVD. This association remained unchanged even after controlling for potential confounding risk factors. Importantly, our study revealed that incorporation of the BNP levels into a model of potential risk factors significantly improved assessment of the likelihood of PAD in T2DM patients. This is the first study providing statistical elucidation of the clinical value of the BNP levels in assessment of the risk of PAD in T2DM outpatients. These findings highlight the potential additive value of measurement of the BNP levels in the risk assessment of PAD in T2DM patients (especially in patients without overt CVD).

These findings have important clinical implications because patients with T2DM have an increased risk of developing PAD. Identifying novel risk factors for PAD may help in the development of strategies for the prevention and treatment of PAD in T2DM patients. Some conventional risk biomarkers (smoking, hypertension, hyperlipidemia, HbA1c level and CRP level) were independently associated with PAD [20-22].

However, traditional risk factors do not entirely explain the excess risk of PAD in some subjects. Monitoring non-conventional risk biomarkers for PAD in T2DM may be particularly important for the prevention and treatment of PAD. BNP levels are known to be elevated in T2DM patients with asymptomatic diastolic dysfunction [23]. It has been acknowledged that subjects with higher BNP levels are more likely to have conventional cardiovascular risk factors (e.g., hypertension [24], ischemic stroke [25,26], and chronic kidney disease [27]). However, BNP levels are independent of cardiac function and cardiovascular factors [28]. BNP release can be stimulated by various factors, including inflammatory cytokines [29] and renal impairment [30]. All of our subjects had a clear diagnosis of T2DM and those with diabetic complications that could affect the metabolism of BNP were excluded or limited. In our study cohort, the high morbidity of PAD was positively correlated with the BNP levels even after adjustment for other risk factors. Subjects in the highest quartile of the BNP level had a 1.25-fold increased risk of developing PAD compared with subjects in the lowest quartile (first quartile) of the BNP levels. There were a significantly increased risk for patients in the third quartile of the BNP levels but not for patients in the second quartile, suggesting a possible threshold effect of the BNP levels for the prediction of PAD. Furthermore, our data showed that a 1-SD increase in the BNP levels were associated with a 1.16-fold increased risk of PAD after adjustment for the known risk factors for PAD. The BNP levels were also inversely correlated with the ABI (*r* = −0.453, *p* = 0.033). It has been suggested that cardiac endocrine function is more greatly activated in PAD patients than in non-PAD patients [31], which may be why one can predict future coronary artery disease or CVD events in PAD patients with higher BNP levels [32]. Higher BNP levels have been associated with lower functional capacity in the vascular system [33]. Hence, consideration of BNP levels is important for improved prediction of PAD in T2DM patients without overt CVD.

The mechanisms through which the BNP levels and PAD are associated are not clear. Studies have shown that the natriuretic peptide family may have a role as anti-migration factors for vascular smooth muscle cells [34]. They also have beneficial effects in T2DM with PAD because natriuretic peptides can promote angiogenesis, modify the function of vascular endothelial cells, reduce cardiac load, and improve blood supply to the legs owing to their diuretic and vasodilatory effects [35-37]. In Wistar rats, pretreatment with BNP can attenuate the excessive production of radical oxygen species [38]. Various studies have indicated that BNP has aprotective role in vascular disease, butour data support the notion that higher BNP levels predispose to PAD development in T2DM. However, thesediffering results may not be contradictory because Kuhn et al. [39] found that BNP is expressed in activated satellite cells within ischemic muscle, and suggested that localized BNP elicited protective endothelial effects. However, because of the impairment of BNP receptors in atherosclerosis or ischemic vascular disease [40], the protective effect of BNP is weakened [41], and BNP levels are increased in response to the severity of ischemia as a protective effect. Nevertheless, the production and secretion of BNP is the result of a complex integration among mechanical, chemical, hemodynamic, humoral, ischemic, and inflammatory inputs in PAD [31,42], and the specific mechanism remains to be elucidated. BNP is likely to be a new therapeutic strategyfor T2DM patients with PAD.

Some potential limitations of our study should be noted. First, BNP levels were measured at a single time point for each patient. Second, we focused on a selected patient cohort hospitalized in an endocrinology department in a single center, and whether the conclusions can be generalized to other institutions requires multicenter studies. Third, the ABI is not the “gold standard” to diagnose PAD. Thus, comparison of BNP levels with the ABI to evaluate PAD due to atherosclerosis may not be an optimal method. Further detailed studies are required to investigate the association between BNP levels and PAD.

## Conclusions

Higher BNP levels (even in the normal range) are associated with a higher prevalence of PAD in T2DM patients. Routine measurement of BNP levels can improve the predictive ability of PAD in T2DM patients.

## Abbreviations

PAD: Peripheral arterial disease; CVD: Cardiovascular disease; BNP: Brain natriuretic peptide; T2DM: Type-2 diabetes mellitus; ABI: Ankle–brachial pressure index; BP: Blood pressure; BMI: Body mass index; TG: Triglyceride; TC: Total cholesterol; HDL-C: High-density lipoprotein-cholesterol; LDL-C: Low-density lipoprotein-cholesterol; CRP: C-reactive protein; HbA1c: Hemoglobin A1c.

## Competing interests

The authors affirm they have no competing interests.

## Authors’ contributions

JQH, CHH, LTL and HCM contributed to the design, analysis and interpretation of this study. HXJ and LQ assisted in the statistical analysis, YWL, XL and ZL contributed to the collection of clinical and laboratory data. JQH contributed to the writing of this manuscript. All authors read and approved the final manuscript.

## Pre-publication history

The pre-publication history for this paper can be accessed here:

http://www.biomedcentral.com/1472-6823/14/27/prepub

## References

[B1] American Diabetes Association Peripheral arterial disease in people with diabetes Diabetes Care 2003 26 3333 3341 14633825 10.2337/diacare.26.12.3333

[B2] Wattanakit K Folsom AR Selvi NE Weatherley BD Pankow JS Brancati FL Hirsch AT Risk factors for peripheral arterial disease incidence in persons with diabetes: The Athe rosclerosis Risk in Communities (ARIC) Study Atherosclerosis 2005 180 389 397 10.1016/j.atherosclerosis.2004.11.024 15910867

[B3] Walters DP Gatling W Mullee MA Hill RD The prevalence, detection, and epidemiological correlates of peripheral vascular disease: a comparison of diabetic and non-diabetic subjects in an English community Diabet Med 1992 9 710 715 10.1111/j.1464-5491.1992.tb01878.x 1395462

[B4] Wannamethee SG Welsh P Lowe GD Gudnason V Di Angelantonio E Lennon L Rumley A Whincup PH Sattar N N-terminal pro-brain natriuretic Peptide is a more useful predictor of cardiovascular disease risk than C-reactive protein in older men with and without pre-existing cardiovascular disease J Am Coll Cardiol 2011 58 56 64 10.1016/j.jacc.2011.02.041 21700090

[B5] Ashley KE Galla JM Nicholls SJ Brain natriuretic peptides as biomarkers for atherosclerosis Prev Cardiol 2008 11 172 176 10.1111/j.1751-7141.2008.08578.x 18607154

[B6] Svensson P de Faire U Niklasson U Hansson LO Ostergren J Plasma NT-pro-BNP concentration is related to ambulatory pulse pressure in peripheral arterial disease Blood Press 2005 14 99 106 10.1080/08037050510008931 16036487

[B7] Dieplinger B Poelz W Haltmayer M Mueller T Association of adiponectin and aminoterminal proBNP in peripheral arterial disease Clin Chim Acta 2007 377 192 197 10.1016/j.cca.2006.09.022 17112494

[B8] Mueller T Dieplinger B Poelz W Endler G Wagner OF Haltmayer M Aminoterminal pro-B-type natriuretic peptide as predictor of mortality in patients with symptomatic peripheral arterial disease: 5-year follow-up data from the linz peripheral arterial disease study Clin Chem 2009 55 68 77 18988753 10.1373/clinchem.2008.108753

[B9] Ye Z Ali Z Klee GG Mosley TH Jr Kullo IJ Associations of candidate biomarkers of vascular disease with the ankle-brachial index and peripheral arterial disease Am J Hypertens 2013 26 495 502 10.1093/ajh/hps073 23467205 PMC3626040

[B10] Salomaa V Havulinna A Saarela O Zeller T Jousilahti P Jula A Muenzel T Aromaa A Evans A Kuulasmaa K Blankenberg S Thirty-one novel biomarkers as predictors for clinically incident diabetes PLoS One 2010 5 e10100 10.1371/journal.pone.0010100 20396381 PMC2852424

[B11] Pfister R Sharp S Luben R Welsh P Barroso I Salomaa V Meirhaeghe A Khaw KT Sattar N Langenberg C Wareham NJ Mendelian randomization study of B-type natriuretic peptide and type 2 diabetes: evidence of causal association from population studies PLoS Med 2011 8 e1001112 10.1371/journal.pmed.1001112 22039354 PMC3201934

[B12] Newton-Cheh C Larson MG Vasan RS Levy D Bloch KD Surti A Guiducci C Kathiresan S Benjamin EJ Struck J Morgenthaler NG Bergmann A Blankenberg S Kee F Nilsson P Yin X Peltonen L Vartiainen E Salomaa V Hirschhorn JN Melander O Wang TJ Association of common variants in NPPA and NPPB with circulating natriuretic peptides and blood pressure Nat Genet 2009 41 348 353 10.1038/ng.328 19219041 PMC2664511

[B13] Expert Committee on the Diagnosis and Classification of Diabetes Mellitus Report of the expert committee on the diagnosis and classification of diabetes mellitus Diabetes Care 2003 26 S5 S20 12502614 10.2337/diacare.26.2007.s5

[B14] Hirsch AT Haskal ZJ Hertzer NR Bakal CW Creager MA Halperin JL Hiratzka LF Murphy WR Olin JW Puschett JB Rosenfield KA Sacks D Stanley JC Taylor LM Jr White CJ White J White RA Antman EM Smith SC Jr Adams CD Anderson JL Faxon DP Fuster V Gibbons RJ Hunt SA Jacobs AK Nishimura R Ornato JP Page RL Riegel B ACC/AHA 2005 Practice Guidelines for the management of patients with peripheral arterial disease Circulation 2006 113 e463 e654 16549646 10.1161/CIRCULATIONAHA.106.174526

[B15] Molitch ME DeFronzo RA Franz MJ Keane WF Mogensen CE Parving HH American Diabetes Association Diabetic nephropathy Diabetes Care 2003 26 S94 S98 12502629 10.2337/diacare.26.2007.s94

[B16] Mancia G Fagard R Narkiewicz K Redon J Zanchetti A Böhm M Christiaens T Cifkova R De Backer G Dominiczak A Galderisi M Grobbee DE Jaarsma T Kirchhof P Kjeldsen SE Laurent S Manolis AJ Nilsson PM Ruilope LM Schmieder RE Sirnes PA Sleight P Viigimaa M Waeber B Zannad F Redon J Dominiczak A Narkiewicz K Nilsson PM Burnier M 2013 ESH/ESC guidelines for the management of arterial hypertension: the Task Force for the Management of Arterial Hypertension of the European Society of Hypertension (ESH) and of the European Society of Cardiology (ESC) Eur Heart J 2013 34 2159 2219 23771844 10.1093/eurheartj/eht151

[B17] McMurray JJ Adamopoulos S Anker SD Auricchio A Böhm M Dickstein K Falk V Filippatos G Fonseca C Gomez-Sanchez MA Jaarsma T Køber L Lip GY Maggioni AP Parkhomenko A Pieske BM Popescu BA Rønnevik PK Rutten FH Schwitter J Seferovic P Stepinska J Trindade PT Voors AA Zannad F Zeiher A ESC Committee for Practice Guidelines ESC Guidelines for the diagnosis and treatment of acute and chronic heart failure 2012: The Task Force for the Diagnosis and Treatment of Acute and Chronic Heart Failure 2012 of the European Society of Cardiology. Developed in collaboration with the Heart Failure Association (HFA) of the ESC Eur Heart J 2012 33 1787 1847 22611136 10.1093/eurheartj/ehs104

[B18] Boulton AJ Vinik AI Arezzo JC Bril V Feldman EL Freeman R Malik RA Maser RE Sosenko JM Ziegler D American Diabetes Association Diabetic neuropathies: a statement by the American diabetes association Diabetes Care 2005 28 956 962 10.2337/diacare.28.4.956 15793206

[B19] Expert Panel on Detection, Evaluation, and Treatment of High Blood Cholesterol in adults Executive summary of the third report of the national cholesterol education program (NCEP) expert panel on detection, evaluation, and treatment of high blood cholesterol in adults (adult treatment panel III) JAMA 2001 285 2486 2497 10.1001/jama.285.19.2486 11368702

[B20] Joosten MM Pai JK Bertoia ML Rimm EB Spiegelman D Mittleman MA Mukamal KJ Associations between conventional cardiovascular risk factors and risk of peripheral artery disease in men JAMA 2012 308 1660 1667 10.1001/jama.2012.13415 23093164 PMC3733106

[B21] Jude EB Eleftheriadou I Tentolouris N Peripheral arterial disease in diabetes – a review Diabet Med 2010 27 4 14 10.1111/j.1464-5491.2009.02866.x 20121883

[B22] Aronow WS Ahn C Weiss MB Babu S Relation of increased hemoglobin A(1c) levels to severity of peripheral arterial disease in patients with diabetes mellitus Am J Cardiol 2007 99 1468 1469 10.1016/j.amjcard.2006.12.085 17493482

[B23] Görmüş U Ozmen D Ozmen B Parildar Z Ozdoğan O Mutaf I Bayindir O Serum N-terminal-pro-brain natriuretic peptide (NT-pro-BNP) and homocysteine levels in type 2 diabetic patients with asymptomatic left ventricular diastolic dysfunction Diabetes Res Clin Pract 2010 87 51 56 10.1016/j.diabres.2009.10.010 19932518

[B24] Phelan D Watson C Martos R Collier P Patle A Donnelly S Ledwidge M Baugh J McDonald K Modest elevation in BNP in asymptomatic hypertensive patients reflects sub-clinical cardiac remodeling, inflammation and extracellular matrix changes PLoS One 2012 7 e49259 10.1371/journal.pone.0049259 23152884 PMC3495762

[B25] Chen X Zhan X Chen M Lei H Wang Y Wei D Jiang X The prognostic value of combined NT-pro-BNP levels and NIHSS scores in patients with acute ischemic stroke Intern Med 2012 51 2887 2892 10.2169/internalmedicine.51.8027 23064562

[B26] Takahashi T Nakamura M Onoda T Ohsawa M Tanno K Itai K Sakata K Sakuma M Tanaka F Makita S Yoshida Y Ogawa A Kawamura K Okayama A Predictive value of plasma B-type natriuretic peptide for ischemic stroke: a community-based longitudinal study Atherosclerosis 2009 207 298 303 10.1016/j.atherosclerosis.2009.04.029 19497572

[B27] Tagore R Ling LH Yang H Daw HY Chan YH Sethi SK Natriuretic peptides in chronic kidney disease Clin J Am Soc Nephrol 2008 3 1644 1651 10.2215/CJN.00850208 18632852 PMC2572269

[B28] Oztekin S Karakurt O Yazıhan N Unal I Relationship of brain natriuretic peptide with metabolic syndrome parameters: an observational study Anadolu Kardiyol Derg 2011 11 678 684 22037102 10.5152/akd.2011.188

[B29] Omland T Advances in congestive heart failure management in the intensive care unit: B-type natriuretic peptides in evaluation of acute heart failure Crit Care Med 2008 36 S17 S27 10.1097/01.CCM.0000296266.74913.85 18158473

[B30] Issa VS Taniguchi LU Park M Cruz LM Bocchi EA Velasco IT Soriano F Positive end-expiratory pressure and renal function influence B-type natriuretic peptide in patients with severe sepsis and septic shock Arq Bras Cardiol 2008 91 107 112 18709262 10.1590/s0066-782x2008001400008

[B31] Clerico A Giannoni A Vittorini S Passino C Thirty years of the heart as an endocrine organ: physiological role and clinical utility of cardiac natriuretic hormones Am J Physiol Heart Circ Physiol 2011 301 H12 H20 10.1152/ajpheart.00226.2011 21551272

[B32] Criqui MH Ninomiya JK Wingard DL Ji M Fronek A Progression of peripheral arterial disease predicts cardiovascular disease morbidity and mortality J Am Coll Cardiol 2008 52 1736 1742 10.1016/j.jacc.2008.07.060 19007695 PMC2871035

[B33] Fan J Jouni H Khaleghi M Bailey KR Kullo IJ Serum N-terminal pro-B-type natriuretic peptide levels are associated with functional capacity in patients with peripheral arterial disease Angiology 2012 63 435 442 10.1177/0003319711423095 22096207 PMC3855435

[B34] Keda M Kohno M Yasunari K Yokokawa K Horio T Ueda M Morisaki N Yoshikawa J Natriuretic peptide family as a novel antimigration factor of vascular smooth muscle cells Arterioscler Thromb Vasc Biol 1997 17 731 736 9108787 10.1161/01.atv.17.4.731

[B35] Chen H Levine YC Golan DE Michel T Lin AJ Atrial natriuretic peptide-initiated cGMP pathways regulate vasodilatorstimulated phosphoprotein phosphorylation and angiogenesis in vascular endothelium J Biol Chem 2008 283 4439 4447 10.1074/jbc.M709439200 18079117

[B36] Park K Itoh H Yamahara K Sone M Miyashita K Oyamada N Sawada N Taura D Inuzuka M Sonoyama T Tsujimoto H Fukunaga Y Tamura N Nakao K Therapeutic potential of atrial natriuretic peptide administration on peripheral arterial diseases Endocrinology 2008 149 483 491 10.1210/en.2007-1094 17991722

[B37] Shmilovich H Ben-Shoshan J Tal R Afek A Barshack I Maysel-Auslander S Harats D Keren G George J B-type natriuretic peptide enhances vasculogenesis by promoting number and func-tional properties of early endothelial progenitor cells Tissue Eng Part A 2009 15 2741 2749 10.1089/ten.tea.2008.0414 19275472

[B38] Talha S Bouitbir J Charles AL Zoll J Goette-Di Marco P Meziani F Piquard F Geny B Pretreatment with brain natriuretic peptide reduces skeletal muscle mitochondrial dysfunction and oxidative stress after ischemia-reperfusion J Appl Physiol 2013 114 172 179 10.1152/japplphysiol.00239.2012 23104692

[B39] Kuhn M Völker K Schwarz K Carbajo-Lozoya J Flögel U Jacoby C Stypmann J van Eickels M Gambaryan S Hartmann M Werner M Wieland T Schrader J Baba HA The natriuretic peptide/guanylyl cyclase–a system functions as a stress-responsive regulator of angiogenesis in mice J Clin Invest 2009 119 2019 2030 10.1172/JCI37430 19487812 PMC2701863

[B40] Tokudome T Kishimoto I Yamahara K Osaki T Minamino N Horio T Sawai K Kawano Y Miyazato M Sata M Kohno M Nakao K Kangawa K Impaired recovery of blood flow after hind-limb ischemia in mice lacking guanylyl cyclase-a, a receptor for atrial and brain natriuretic peptides Arterioscler Thromb Vasc Biol 2009 29 1516 1521 19628785 10.1161/ATVBAHA.109.187526

[B41] Schirger JA Grantham JA Kullo IJ Jougasaki M Wennberg PW Chen HH Lisy O Miller V Simari RD Burnett JC Jr Vascular actions of brain natriuretic peptide: modulation by atherosclerosis and neutral endopeptidase inhibition J Am Coll Cardiol 2000 35 796 801 10.1016/S0735-1097(99)00593-8 10716485

[B42] Gruden G Barutta F Chaturvedi N Schalkwijk C Stehouwer CD Pinach S Manzo M Loiacono M Tricarico M Mengozzi G Witte DR Fuller JH Perin PC Bruno G NH2-terminal probrain natriuretic peptide is associated with diabetes complications in the EURODIAB Prospective Complications Study: the role of tumor necrosis factor-α Diabetes Care 2012 35 1931 1936 10.2337/dc12-0089 22699286 PMC3425012

